# Current Strategies of Antiviral Drug Discovery for COVID-19

**DOI:** 10.3389/fmolb.2021.671263

**Published:** 2021-05-13

**Authors:** Miao Mei, Xu Tan

**Affiliations:** ^1^Tsinghua-Peking Center for Life Sciences, Beijing Advanced Innovation Center for Structural Biology, Beijing Frontier Research Center for Biological Structure, MOE Key Laboratory of Bioorganic Phosphorus Chemistry & Chemical Biology, School of Pharmaceutical Sciences, Center for infectious Disease Research, School of Medicine, Tsinghua University, Beijing, China; ^2^Beijing Advanced Innovation Center for Structural Biology, Beijing Frontier Research Center for Biological Structure, MOE Key Laboratory of Bioorganic Phosphorus Chemistry & Chemical Biology, School of Pharmaceutical Sciences, Center for Infectious Disease Research, School of Medicine, Tsinghua University, Beijing, China

**Keywords:** COVID-19, SARS-CoV-2, direct-acting antiviral, host-targeting antiviral, high throughput screening, artificial intelligence, antibody, immuno-regulator

## Abstract

SARS-CoV-2 belongs to the family of enveloped, single-strand RNA viruses known as Betacoronavirus in Coronaviridae, first reported late 2019 in China. It has since been circulating world-wide, causing the COVID-19 epidemic with high infectivity and fatality rates. As of the beginning of April 2021, pandemic SARS-CoV-2 has infected more than 130 million people and led to more than 2.84 million deaths. Given the severity of the epidemic, scientists from academia and industry are rushing to identify antiviral strategies to combat the disease. There are several strategies in antiviral drugs for coronaviruses including empirical testing of known antiviral drugs, large-scale phenotypic screening of compound libraries and target-based drug discovery. To date, an increasing number of drugs have been shown to have anti-coronavirus activities *in vitro* and *in vivo*, but only remdesivir and several neutralizing antibodies have been approved by the US FDA for treating COVID-19. However, remdesivir’s clinical effects are controversial and new antiviral drugs are still urgently needed. We will discuss the current status of the drug discovery efforts against COVID-19 and potential future directions. With the ever-increasing movability of human population and globalization of world economy, emerging and reemerging viral infectious diseases seriously threaten public health. Particularly the past and ongoing outbreaks of coronaviruses cause respiratory, enteric, hepatic and neurological diseases in infected animals and human (Woo et al., 2009). The human coronavirus (HCoV) strains (HCoV-229E, HCoV-OC43, HCoV-NL63, and HCoV-HKU1) usually cause common cold with mild, self-limiting upper respiratory tract infections. By contrast, the emergence of three deadly human betacoronaviruses, middle east respiratory syndrome coronavirus (MERS) (Zaki et al., 2012), severe acute respiratory syndrome coronavirus (SARS-CoV) (Lee et al., 2003), the SARS-CoV-2 (Jin et al., 2020a) highlight the need to identify new treatment strategies for viral infections. SARS-CoV-2 is the etiological agent of COVID-19 disease named by World Health Organization (WHO) (Zhu N. et al., 2020). This disease manifests as either an asymptomatic infection or a mild to severe pneumonia. This pandemic disease causes extent morbidity and mortality in the whole world, especially regions out of China. Similar to SARS and MERS, the SARS CoV-2 genome encodes four structural proteins, sixteen non-structural proteins (nsp) and accessory proteins. The structural proteins include spike (S), envelope (E), membrane (M), nucleoprotein (N). The spike glycoprotein directly recognizes and engages cellular receptors during viral entry. The four non-structural proteins including papain-like protease (PL^pro^), 3-chymotrypsin-like protease (3CL^pro^), helicase, and RNA-dependent RNA polymerase (RdRp) are key enzymes involved in viral transcription and replication. The spike and the four key enzymes were considered attractive targets to develop antiviral agents (Zumla et al., 2016). The catalytic sites of the four enzymes of SARS-CoV2 share high similarities with SARS CoV and MERS in genomic sequences (Morse et al., 2020). Besides, the structures of the key drug-binding pockets are highly conserved among the three coronaviruses (Morse et al., 2020). Therefore, it follows naturally that existing anti-SARS-CoV and anti-MERS drugs targeting these enzymes can be repurposed for SARS-CoV-2. Based on previous studies in SARS-CoV and MERS-CoV, it is anticipated a number of therapeutics can be used to control or prevent emerging infectious disease COVID-19 (Li and de Clercq, 2020; Wang et al., 2020c; Ita, 2021), these include small-molecule drugs, peptides, and monoclonal antibodies. Given the urgency of the SARS-CoV-2 outbreak, here we discuss the discovery and development of new therapeutics for SARS-CoV-2 infection based on the strategies from which the new drugs are derived.

## Empirically Repositioning of Existing Antiviral Drugs Against COVID-19

Currently, there is no highly efficacious and specific treatment for SARS-CoV-2. Therefore, it is urgent need to identify effective antiviral agents from existing drugs to combat the infection. These drugs can be rapidly repurposed to clinic application for treating COVID-19 patients given their proven safety. These compounds include direct acting antivirals (Table 1) and host targeting antiviral agents (Table 2).

**TABLE 1:** Representative direct-acting antivirals for CoV infections.

**Name of drugs**	**Mechanism of action**	***In vitro***	***In vivo***	**Clinical trial**	**Approved**	**References**
EK1C4	Mediates membrane fusion by targeting the 6-HB of HIV and the HR1 domain in divergent human coronaviruses	HIV, SARS-CoV-2	HIV, SARS-CoV-2	N.A.	N.A.	Xia et al., 2020a
Arbidol	Impedes spike trimerization	Influenza, HBV, HCV, CHIKV, RSV, reovirus, Hantaan virus, coxsackie virus B5 ZIKV, SARS-CoV-2	Influenza, SARS-CoV, SARS-CoV-2	Influenza, SARS-CoV, SARS-CoV-2	Influenza	Blaising et al., 2014; Nojomi et al., 2020; Vankadari, 2020

**Viral RNA: Nucleoside/nucleotide analogs**						
Favipiravir	Incorporates into the genome of RNA viruses to interrupt viral RNA synthesis by blocking RdRp activity	Influenza, EBOV, MARV, YFV, RVFV JUNV, LASV, CCHFV, CHIKV, WNV, norovirus and enterovirus, arenaviruses, Nipah virus, SARS-CoV-2	Influenza, Ebola, SARS-CoV-2	Ebola,SARS-CoV-2	Influenza	Sissoko et al., 2016; Furuta et al., 2017; de Clercq, 2019
Ribavirin	Incorporates into the genome of RNA viruses to interrupt viral RNA synthesis by blocking RdRp activity	HCV, RSV, SARS-CoV, MERS-CoV, SARS-CoV-2, viral haemorrhagic fevers	HCV, RSV, SARS-CoV, MERS-CoV, SARS-CoV-2	HCV, RSV, SARS-CoV, MERS-CoV, SARS-CoV-2	HCV, RSV	Zumla et al., 2016
Remdesivir	Incorporates into the genome of RNA viruses to interrupt viral RNA synthesis by blocking RdRp activity	Bat CoVs, SARS-CoV, MERS-CoV, SARS-CoV-2	Bat CoVs, SARS-CoV, MERS-CoV, SARS-CoV-2	SARS-CoV, and MERS-CoV, SARS-CoV-2	HIV, Ebola, SARS-CoV-2	Warren et al., 2016; Sheahan et al., 2017; Mulangu et al., 2019; Caly et al., 2020; Wang et al., 2020c
Galidesivir	Incorporates into the genome of RNA viruses to interrupt viral RNA synthesis by blocking RdRp activity	ZIKV SARS-CoV MERS- CoV SARS-CoV-2	ZIKV SARS- CoV MERS- CoV SARS-CoV-2	Yellow fever, SARS- CoV, MERS-CoV, SARS-CoV-2	HCV	Elfiky, 2020; Lim et al., 2020
EIDD-2801	Incorporates into the genome of RNA viruses to interrupt viral RNA synthesis by blocking RdRp activity	SARS-CoV-2	SARS- CoV, MERS- CoV, SARS-CoV-2	EIDD-2801	N.A.	Sheahan et al., 2020; Wahl et al., 2021

**Viral enzyme: protease inhibitors**						
Disulfiram	Inhibits Papain-like protease	MERS SARS, SARS-CoV-2	N.A.	N.A.	Alcohol dependence	Lin et al., 2018; Ma et al., 2020
Lopinavir- ritonavir	Inhibits 3 chymotrypsin-like protease	SARS-CoV, MERS-CoV, SARS-CoV-2 HCoV-229E, HCoV-NL63, animal CoVs	SARS-CoV, MERS-CoV, SARS-CoV-2, HCoV-229E, HCoV-NL63, animal CoVs	SARS-CoV, MERS-CoV, SARS-CoV-2	HIV	Zumla et al., 2016; Cao B. et al., 2020
Darunavir	Inhibits dimerization of HIV-1 protease	SARS-CoV-2	N.A.	SARS-CoV-2	HIV1	Chen J. et al., 2020; WHO Solidarity Trial Consortium et al., 2021
N3	Inhibits M^pro^	SARS-CoV-2	N.A.	N.A.	N.A.	Jin et al., 2020b
11a/11b	Inhibits M^pro^	SARS-CoV-2	SARS-CoV-2	N.A.	N.A.	Dai et al., 2020
Carmofur	Inhibits M^pro^	SARS-CoV-2	N.A.	N.A.	N.A.	Jin et al., 2020c

*EBOV, Ebola virus, MARV, Marburg virus, dengue virus, JUNV, Junin virus, CCHFV, Crimean-Congo hemorrhagic fever virus, RVFV, Rift Valley fever virus, LASV, Lassa virus, YFV, yellow fever virus, RSV, respiratory syncytial virus, CHIKV, Chikungunya virus.*

**TABLE 2:** Representative host-targeting antivirals for CoV infections.

**Name of drugs**	**Mechanism of action**	***In vitro***	***In vivo***	**Clinical trial**	**Approved**	**References**
Glycyrrhizin	Blocks the binding of ACE2 and RBD	SARS-CoV-2	N.A.	SARS-CoV-2(NCT04487964)	N.A.	Zhou and Huang, 2020
Nobiletin	Blocks the binding of ACE2 and RBD	SARS-CoV-2	N.A.	N.A.	N.A.	Zhou and Huang, 2020
Neohesperidin	Blocks the binding of ACE2 and RBD	SARS-CoV-2	N.A.	N.A.	N.A.	Zhou and Huang, 2020
SSAA09E2	Blocks the binding of ACE2 and RBD	SARS-CoV-2	N.A.	N.A.	N.A.	Oany et al., 2020; Oroojalian et al., 2020
Chlorpromazine	Inhibits endocytosis	MERS-CoV, SARS-CoV-2	N.A.	SARS-CoV-2(NCT04516512)	N.A.	Glebov, 2020
Chloroquine/hydroxychloroquine	Immunoregulater	HIV, SARS, Influenza, SARS-CoV-2	HIV, SARS, Influenza, SARS-CoV-2	HIV, SARS, Influenza, SARS-CoV-2	Anti-malarial	Cao Y. et al., 2020; Ferner and Aronson, 2020; Touret and de Lamballerie, 2020; Wang et al., 2020c
Camostat Mesylate	TMPRSS2 inhibitor	SARS-CoV, MERS-CoV, SARS-CoV-2	Influenza, SARS-CoV, MERS-CoV, SARS-CoV-2	SARS-CoV-2	N.A.	Hoffmann et al., 2020a; Breining et al., 2021
Nafamostat	Serine proteinase inhibitor	MERS-CoV, SARS-CoV-2	N.A.	N.A.	N.A.	Yamamoto et al., 2016; Wang et al., 2020c
Ivermectin	N.A.	Influenza, DENV, SARS-CoV-2, VEEV	ZIKV	SARS-CoV-2	Anti-parasitic agent	Caly et al., 2020; Rajter et al., 2021
Nitazoxanide	Boosts host innate immune responses and tackle cytokine storm	Human and animal coronaviruses including SARS-CoV-2	N.A.	N.A.	Antiprotozoal agent	Wang et al., 2020c; Rajter et al., 2021
Interferon alfa-2a and -2b	Stimulates innate antiviral responses	HBV, HCV, SARS-CoV-2	N.A.	SARS-CoV-2	HBV,HCV	Zeng Y.M. et al., 2020; WHO Solidarity Trial Consortium et al., 2021
Deguelin	PI3K/Akt inhibitor	HCV, HCMV, SARS-CoV-2	N.A.	N.A.	Cancer	Nukui et al., 2018; Liao et al., 2020; Sun et al., 2021
Nilotinib	BCR-ABL inhibitor, induces autophagy by activating AMPK	SARS-CoV-2	N.A.	N.A.	Chronic myeloid leukemia	Sun et al., 2021
Sorafenib	Multikinase inhibitor of Raf-1 and B-Raf	SARS-CoV-2	N.A.	N.A.	Hepatocellular carcinoma	Sun et al., 2021

*Venezuelan Equine Encephalitis Virus (VEEV), Human Cytomegalovirus (HCMV).*

### Direct-Acting Antivirals

The functional domains of the spike protein of SARS-CoV-2 are highly conserved with those of SARS-CoV (Xia et al., 2020b). These domains can be classified into S1 (aa 14-685) and S2 (aa 686-1273) subunit. S1 subunit includes the N-terminal domain (NTD), the receptor-binding domain (RBD), and the receptor-binding motif (RBM), while the S2 subunit consists of the fusion peptides, heptad repeat regions (HR1, HR2), the transmembrane domain (TMD) and the cytoplasm domain (CD). HR1 and HR2, each having three alpha helical segments, pack together to form the six-helical bundle (6-HB). This 6-HB structure is similar to that of HIV envelope protein in the form as well as the function, which is to facilitate virus entry into the cells. Based on the development of HIV fusion peptide inhibitor targeting the 6-HB of HIV, EK1, a peptide-based fusion inhibitor, was developed to target the HR1 domain in divergent human coronaviruses. EK1C4, modified by adding PEG and cholesterol moieties to the EK1 peptide, more potently inhibited SARS-CoV-2 spike-mediated membrane fusion and pseudovirus infection (IC50 = 15.8 nM for inhibition of SARS-CoV-2 pseudovirus). Its inhibitory ability is highly effective against multiple coronaviruses including SARS-CoV and SARS-CoV-2 in human cells and mouse models with low or no toxicity to the hosts (Xia et al., 2020a), thereby representing a pan-coronavirus inhibitor that potentially applies to strain variants of SARS-CoV-2 and coronaviruses that might emerge in the future.

Arbidol, an approved anti-influenza drug, inhibits early stage of viral replication by blocking the virus fusion with the cell membrane, including SARS-CoV-2 (Wu et al., 2020a). It can effectively inhibit SARS-CoV-2 infection *in vitro* via impeding spike trimerization (Vankadari, 2020) and blocking the spike/ACE2 interaction (Kadam and Wilson, 2017). Several clinical trials (IRCT20180725040596N2, NCT04260594) have demonstrated that arbidol has some effect in reducing the duration of hospitalization (Nojomi et al., 2020) and mortality of COVID-19 patients (Wang et al., 2020g). Arbidol monotherapy significantly inhibited SARS-CoV-2 viral load compared with lopinavir/ritonavir (Zhu Z. et al., 2020). Arbidol combined with LPV/r is superior to LPV/r alone (Chen C. et al., 2020). Compared with dual combination antiviral tests, the triple combination antiviral therapy of arbidol, lopinavir/litonavir and rIFNα-2b showed better therapeutic efficacy (Wei et al., 2020). However, in another clinical trial, combination with arbidol and favipiravir did not change the clinical recovery rate (Chen C. et al., 2020).

Nucleoside/nucleotide analogs (NAs), which belongs to adenine or guanine derivatives, block viral RNA synthesis by targeting viral RdRp and in a broad spectrum of RNA viruses, including human coronaviruses, human immunodeficiency virus (HIV), hepatitis B virus (HBV) and hepatitis C virus (HCV). Up to now, NAs favipiravir, ribavirin, remdesivir, galidesivir, sofosbuvir, tenofovir, NHC (β-DN4-hydroxycytidine, EIDD-1931) and EIDD-2801 have potential to treat SARS-CoV-2 (Elfiky, 2020; Sheahan et al., 2020; Wahl et al., 2021), since they tightly bind to RdRp of SARS-CoV-2 by molecular docking analysis (Elfiky, 2020). Favipiravir, an approved pyrazinecarboxamide derivative against influenza virus, can selectively and effectively inhibit the RdRp activity of RNA viruses such as influenza virus, Ebola virus, yellow fever virus, chikungunya virus, norovirus and enterovirus (Furuta et al., 2017; de Clercq, 2019). It could also block SARS CoV-2 *in vitro* (Wang et al., 2020c). Patients infected with SARS-CoV-2 were recruited in randomized trials to evaluate the efficacy and safety of favipiravir alone (Doi et al., 2020; Udwadia et al., 2021) or in combination with other drugs (ChiCTR2000029544) (Tu et al., 2020). Several of these trials have identified modest effect of favipiravir in shortening the time to recovery of COVID-19 patients.

Ribavirin, a guanine derivative approved in combiantion with other anti-medication for treating HCV and respiratory syncytial virus (RSV), has been evaluated in patients with SARS and MERS, but some patients may manifest side effects such as severe anemia at high doses (Zumla et al., 2016) and we do not know whether it sufficiently blocks SARS-CoV-2 (Eslami et al., 2020).

Remdesivir is a phosphoramidate prodrug of an adenine derivative and a broad-spectrum antiviral medication against pathogenic animal and human coronaviruses: bat CoVs, SARS-CoV, and MERS-CoV infection *in vitro* and *in vivo* (Sheahan et al., 2017; Warren et al., 2016). Its chemical structure is similar to that of tenofovir alafenamide, an approved HIV reverse transcriptase inhibitor. Remdesivir has been tested in a clinical for Ebola virus infected patients but showed no beneficial effect for reducing death rates compared with the control groups treated with different antibody therapies (Mulangu et al., 2019). The mechanism of action of redemsivir is that it is incorporated into nascent viral RNA chains, which results in pre-mature termination of the RNA replication by the RdRp of RNA viruses (Warren et al., 2016). Remdesivir demonstrated a potent anti-SARS-CoV-2 activity with high selectivity index values (Wang et al., 2020c). There are several successful cases of remdesivir in treating COVID-19, for example, the reported patients with mild to moderate COVID-19 were given a medication of intravenous remdesivir, and their clinical symptoms had been recovered (Grein et al., 2020). A US patient with SARS-CoV-2 recovered after receiving intravenous remdesivir in January 2020 (Holshue et al., 2020). Rhesus macaques were challenged with SARS-CoV-2 and treated with remdesivir, unlike the placebo group, macaques treated with remdesivir did not represent the severe disease observed in some patients with COVID-19 (Williamson et al., 2020). Several phase III trials were initiated in early February 2020 to evaluate the efficacy and safety of intravenous remdesivir in patients with SARS-CoV-2 (NCT04252664, NCT04257656, ISRCTN83971151, NCT04315948) (Goldman et al., 2020; Wang et al., 2020f). However, results from these trials produced mixed results which include remdesevir partially improved the symptom of wild to moderate patients or there are not significant difference in treated and untreated patients. Remdesivir, in combination of the Janus kinase inhibitor baricitinib (Kalil et al., 2021), has been granted emergency use authorization for COVID-19 by US Food and Drug Administration (FDA).

Galidesivir, an approved adenosine analog against HCV, is subsequently developed as broad-spectrum antiviral agent against yellow fever virus, Ebola virus, Zika virus, SARS and MERS-CoVs (Zumla et al., 2016). Structure-based modeling suggests that galidesivir binds to the non-catalytic site of SRAS-CoV-2 RdRp, therefore it might exert an allosteric inhibition on RdRp (Mishra and Rathore, 2021).

EIDD-2801, a prodrug of a broad-spectrum anti-CoV inhibitor NHC, a ribonucleoside analog, blocked SARS-CoV-2 infection *in vitro* and *in vivo* (Sheahan et al., 2020; Wahl et al., 2021). It also promoted pulmonary function and reduced virus titer and body weight loss of mice infected with SARS-CoV and MERS-CoV (Sheahan et al., 2020). Particularly, in human lung-only mice (LoM), which mimic the condition of human lung in a physiological context, treatment or prophylaxis administration (only one dose) of EIDD-2801 potently inhibited SARS-CoV-2 infection and pathogenesis (Wahl et al., 2021). Currently, prophylactic administration of EIDD-2801 is being tested in phase II/III clinical trial for treating COVID-19 (NCT04405570 and NCT04405739) (Cox et al., 2021).

Several approved viral protease inhibitors including disulfiram, lopinavir, and ritonavir and darunavir, are widely used to treat HIV-1 and HCV infection by selectively inhibiting viral proteases and their cleavage. Thus they have potential to inhibit coronavirus infection. Disulfiram, an alcoholism averting drug with low adverse effects, inhibited the PL^pro^ of MERS and SARS *in vitro* (Lin et al., 2018). It has been reported as a non-specific M^pro^ inhibitor (Ma et al., 2020). But the clinical efficacy of disulfiram remains to be demonstrated. HIV protease inhibitors lopinavir and ritonavir have been shown to have no benefit for hospitalized adult patients with severe COVID-19 in a trial (Cao B. et al., 2020). Lopinavir and ritonavir improved clinical symptoms of patients with SARS in a non-randomized open-label trial (Zumla et al., 2016). HIV protease belongs to the aspartic protease family, whereas the two coronavirus proteases are from the cysteine protease family. To date, we do not know whether HIV protease inhibitors could effectively inhibit the 3CL^pro^ and PL^pro^ of SARS CoV-2. Furthermore, HIV protease inhibitors specifically fit the C2 symmetric pocket in the catalytic site of the HIV protease dimer, but this pocket is absent in coronavirus proteases. Darunavir, a second-generation anti-HIV-1 protease inhibitor, can inhibit SARS-CoV-2 replication *in vitro*, but darunavir plus cobicistat did not promote viral clearance compared with IFNα 1b treatment alone in treating COVID-19 (NCT04252274) (Chen J. et al., 2020). Darunavir or lopinavir, in combination with hydroxychloroquine, caused electrocardiogram abnormality in patients with history of cardiovascular diseases (Meriglier et al., 2021). It indicated that this combination is not safe for this group of subjects. Furthermore, in a WHO SOLIDARITY trial, remdesivir, hydroxychloroquine, lopinavir and interferon regimens appeared to have little or no effect on hospitalized COVID-19 (WHO Solidarity Trial Consortium et al., 2021).

Currently, multiple small molecules-based on structures have been identified with promisingly inhibitory effects against SARS-CoV-2. Except for RdRp as an ideal antiviral target, the main protease (M^pro^) is also reported as an attractive target for inhibiting viral replication and transcription. Currently known M^pro^ inhibitor-based structures include N3, 11a, 11b, Camostat Mesylate, Carmofur. The crystal structure of inhibitors-M^pro^ complex has been reported (Wang et al., 2020d). N3, which is identified by computer-aided drug design, is a potent irreversible inhibitor of main protease and inserts into the substrate-binding pocket of M^pro^ according to structural analysis (Jin et al., 2020b). Dai et al. (2020) designed and synthesized two lead compounds 11a and 11b, which inhibited SARS-CoV-2 infection with low toxicity *in vitro* and *in vivo*, and with excellent pharmacokinetic properties covalently binding to the catalytic site of SARS-CoV-2 M^pro^ (Dai et al., 2020). In addition, combination of multiple techniques identified six M^pro^ inhibitors including Ebselen, Disulfiram, Tideglusib, Carmofur, Shikonin, PX-12, which inhibited enzymatic activity of PL^pro^ and M^pro^ of SARS-CoV-2 and presented non-specifical anti-SARS-CoV-2 activity *in vitro*. The catalytic cysteine 145 of M^pro^ was covalently bound to 11a or 11b (Dai et al., 2020) or carmofur (Jin et al., 2020c).

### Host-Targeting Antivirals

Small-molecule agents approved for other human diseases may modulate the virus–host interactions. Currently there are very few host-targeting small molecule drugs approved for antiviral purpose, with the HIV entry inhibitor maraviroc as a notable example (de Clercq and Li, 2016). Given the exploding information on the virus-host interaction, it is expected that host-targeting small molecules are the next frontier of antiviral drug discovery. Obvious advantages of these host-targeting drugs are broad-spectrum and less likelihood of drug resistance.

SARS-CoV-2 entering into host cells is key step of its life cycle, so blocking this step is critical for prevention of virus infection. Angiotensin-converting enzyme 2 (ACE2), which is highly expressed in lung, small intestine, brain, testis, kidney (Verdecchia et al., 2020), is the functional receptor of NL63 (Milewska et al., 2018), SARS-CoV (Li et al., 2003) and SARS-CoV-2 (Hoffmann et al., 2020a; Wang et al., 2020e) to facilitate their entry. ACE2 from human, monkey, pig, civet cells promotes cellular entry of SARS-CoV-2 when overexpressed, the result indicates that it is a common receptor for SARS-CoV-2 infection in these hosts (Zhou et al., 2020a). The RBD in SARS-CoV-2 spike directly binds with ACE2, thus the interaction of spike/ACE2 can be interrupted by neutralizing antibodies and small molecules. Several natural compounds, including baicalin, scutellarin, nicotianamine, and glycyrrhizin have potential to block attachment and entry of SARS-CoV-2 (Chen and Du, 2020). Glycyrrhizin, nobiletin, and neohesperidin that bind to ACE2 can partially block the binding of ACE2 and RBD (Zhou and Huang, 2020). The entry inhibitors in clinical trials have been reviewed (Oroojalian et al., 2020), which includes SSAA09E2 that blocks the interaction of ACE2-RBD. Different viruses may use the same cellular endocytic pathways to target viral entry at the point endocytosis. This strategy is promising for developing broad-spectrum antiviral drugs. There are a variety of approved drugs may have ability to block SARS-CoV-2 endocytosis *in vitro* (Glebov, 2020), including chlorpromazine, fluvoxamine, sertraline, promethazine, nystatin, amiloride, vinblastine, itraconazole, flubendazole, terfenadine, imipramine, beta-methyl cyclodextrin. Among them, chloroquine is a potential broad-spectrum antiviral drug against multiple virus infections (Savarino et al., 2006a; Yan et al., 2013) and widely used as anti-malarial, anti-cancer inhibitor (Savarino et al., 2006b), as well as an approved immune modulator for autoimmune diseases. Chloroquine is used for the treatment of COVID-19 as it inhibits the spread of SARS-CoV-2 *in vitro* (Ferner and Aronson, 2020; Touret and de Lamballerie, 2020; Wang et al., 2020c) likely by blocking endosomal acidification required for virus/cell fusion. However, chloroquine did not inhibit SARS-CoV-2 infection in human lung adenocarcinoma cell line Calu-3 overexpressing TMPRSS2 (Hoffmann et al., 2020b). An open-label trial (ChiCTR2000029609), a randomized clinical trial (NCT04323527) (Borba et al., 2020) and WHO SOLIDARITY trial were carried out to evaluate the safety and efficacy of chloroquine diphosphate against COVID-19 caused by SARS-CoV-2. The results suggest that chloroquine have little or no effect on hospitalized patients with COVID-19 (Ektorp, 2020).

Two mucosa-specific transmembrane serine protease 2 (TMPRSS2) and 4 (TMPRSS4) catalyze the cleavage of SARS-CoV-2 spike and trigger virus entry into host cells (Zang et al., 2020). Particularly, TMPRSS2 facilitates the spread and pathogenesis of SARS-CoV-2, providing a potential target for antiviral intervention. A clinical TMPRSS2 inhibitor Camostat Mesylate, potently inhibited SARS-CoV and MERS-CoV, also excellently blocked SARS-CoV-2 entry in lung adenocarcinoma cell line Calu-3 at concentrations without obvious toxicity (Hoffmann et al., 2020a). Nafamostat, a serine proteinase inhibitor, potently inhibits MERS-CoV membrane fusion relying on the activity of TMPRSS2 (Yamamoto et al., 2016) and SARS-CoV-2 infection (Wang et al., 2020c).

Ivermectin, an FDA-approved broad spectrum anti-parasitic agent (Gonzalez Canga et al., 2008),is a broad-spectrum antiviral agent with activities against flaviviruses, influenza virus and HIV-1 (Mastrangelo et al., 2012; Wagstaff et al., 2012; Lundberg et al., 2013; Tay et al., 2013; Gotz et al., 2016; Ketkar et al., 2019). It also demonstrated inhibitory effect on SARS CoV-2 infection *in vitro* (Caly et al., 2020). Ivermectin administration significantly reduced the mortality of COVID-19 patients (Rajter et al., 2021). Nitazoxanide, a commercial antiprotozoal agent with an antiviral potential against a broad range of viruses including human and animal coronaviruses, can also block SARS-CoV-2 replication at low micromolar concentration *in vitro* (Wang et al., 2020c). Although the mechanisms of actions of ivermectin and nitazoxanide are still unclear, their broad-spectrum activities suggest host-targeting mechanisms. Further *in vivo* and clinical evaluations of these drugs are warranted.

Pegylated interferon alfa-2a and - 2b alone and in combination with other antiviral agents, approved for treating HBV and HCV infection, could be used to stimulate innate antiviral responses in COVID-19 patients. One research showed that type I and type III IFN have potential inhibitory effects on SARS-CoV-2 *in vitro* (Felgenhauer et al., 2020). However, several clinical trials involving interferons showed that interferon regimens have little or no effect on hospitalized COVID-19 patients (ChiCTR2000029387, ISRCTN83971151, NCT04315948) (Zeng Y.M. et al., 2020; WHO Solidarity Trial Consortium et al., 2021).

Host RNA binding proteins are emerging host factors that are involved in the life cycle of viruses. Recently, Sun et al. reported *in vivo* genomic RNA structure landscape of SARS-CoV-2, and validated several key structure elements influencing viral protein translation and abundance of virus sub-genomic structure. Using deep learning algorithm based on the information of the viral genomic structure, they predicted 42 host binding proteins on SARS-CoV-2 RNA, such as DDX24, NPM1. Further, they identified antisense oligonucleotides (ASOs) targeting viral RNA structure units and FDA-approved drugs Deguelin, Nilotinib, and Sorafenib inhibiting the predicted RNA binding protein expression and showed these compounds decreased SARS-CoV-2 infection in human cells (Sun et al., 2021).

### Traditional Chinese Medicine

Traditional Chinese medicine including plant extracts present potential anti-coronavirus ability for therapeutic development (Li et al., 2005; Park et al., 2017). The active components from traditional Chinese herbs can treat COVID-19 by controlling virus infection and regulating immune response, inflammatory reaction and hypoxia response (Zhang et al., 2020). Combination of Chinese herbs (Lianhuaqingwen capsule) and western medicines have been encouraged to treat infected patients in hospital with good efficacy in China (Xia et al., 2020c). One review summarized natural products in the role of anti-SARS-CoV-2 infection (Zhou and Huang, 2020), including viral targets, natural components and mechanism of action against SARS-CoV-2.

## High-Throughput Screening for Anti-SARS-CoV-2 Drugs

Large scale repurposing screening of known or approved drugs could significantly accelerate the deployment of novel therapies for COVID-19 (Table 3). One of such efforts is profiling FDA-approved small molecules LOPAC-1280 and ReFRAME (Repurposing, Focused Rescue, and Accelerated Medchem) drug library containing about 12,000 bioactive molecules (Riva et al., 2020). A total of 21 known drugs showed inhibition of SARS CoV-2 replication in dose-dependent manners. All of the PIKfyve kinase inhibitor apilimod, the cysteine protease inhibitors MDL-28170 and ONO 5334 presented an efficacious potency against SARS-CoV-2 replication in iPSC cells and apilimod efficiently inhibited SARS-CoV-2 infection in human lung tissues.

**TABLE 3:** Representative high throughputing screened strategies for CoV infections.

**Name of drugs**	**Mechanism of action**	***In vitro***	***In vivo***	**Clinical trial**	**Approved**	**References**
Apilimod	PIKfyve kinase inhibitor	SARS-CoV-2	N.A.	SARS-CoV-2 (NCT04446377)	N.A.	Riva et al., 2020
MDL-28170	The cysteine protease inhibitor	SARS-CoV-2	N.A.	N.A.	N.A.	Riva et al., 2020
ONO 5334	The cysteine protease inhibitor	SARS-CoV-2	N.A.	N.A.	N.A.	Riva et al., 2020
Imatinib	Protein-tyrosine kinase inhibitor, lipid signaling, synthesis and metabolism, blocks the interaction of ACE2-Spike	SARS-CoV-2	N.A.	N.A.	Chronic myeloid leukemia and malignant gastrointestinal stromal tumors	Han et al., 2021
Mycophenolic acid	Inhibitor of IMPDH and guanine monophosphate synthesis	MERS-CoV, HBV, HCV, arboviruses (JEV, WNV, YFV, dengue virus and CHIKV) SARS-CoV-2	MERS-CoV	N.A.	Prevention of rejection after organ transplantation	Han et al., 2021
Quinacrine Dihydrochloride	Inhibits NF-κB and activates p53 signaling, lipid signaling, synthesis and metabolism, blocks the interaction of ACE2-Spike	SARS-CoV-2	N.A.	N.A.	Anti-malarial	Han et al., 2021

In another screening effort, lung and colonic organoid model derived from human pluripotent stem cells were used as the infection target, presumably more physiologically relevant than using cancer cell lines. From this screening, imatinib, mycophenolic acid and quinacrine dihydrochloride were identified from FDA-approved library as SARS-CoV-2 entry inhibitors at physiologically relevant concentrations. Imatinib and QNHC bind with ACE2 with a high affinity based on surface plasmon resonance binding analysis. Imatinib is related to lipid signaling, synthesis and metabolism based on RNA-Seq analysis, therefore likely affects SARS-CoV-2 by modulating host responses (Han et al., 2021).

## Artificial Intelligence-Based Drug Discovery for Anti-SARS-CoV-2

In the big data era, artificial intelligence (AI) algorithms are increasingly being applied for rapid and cost-effective drug discovery (Fleming, 2018). The scale and efficiency that AI brings to drug discovery is especially relevant for treating COVID-19 epidemic. Thus, it supplies a good opportunity for drug discovery and development via introducing AI tools and network medicine, for example, drug target identification based on protein-protein interactome (Zhou et al., 2020d). In one such studies, 332 interactions have been identified between SARS-CoV-2 proteins and human host proteins including ACE2, Furin, TEPRSS2, NRP1, eEF1A, etc. (Gordon et al., 2020). Among of the identified host proteins, 69 druggable targets were selected and two inhibitors can potently block SARS-CoV-2 infection, one by inhibiting mRNA translation (Zotatifin) and the other by regulating sigma-2 and sigma-2 receptors (haloperidol). Also derived from this study, the inhibitor plitidepsin targeting host protein eEFIA, which interacts with multiple coronavirus proteins, potently inhibits SARS-CoV-2 infection *in vivo* (White et al., 2021). From another study, 16 potential anti-coronavirus drugs (Irbesartan, toremifene, camphor, equilin, mesalazine, mercaptopurine, paroxetine, sirolimus, carvedilol, colchicine, dactinomycin, melatonin, quinacrine, eplerenone, emodin, Oxymetholone) were found to inhibit SARS-CoV-2 infection based on drug targets and viral-host interactions through implementing a systemic network medicine platform (Zhou et al., 2020c). Among of them, toremifene, an approved selective estrogenic receptor modulator for treating breast cancer, can inhibit various viral infection including MERS-CoV (Cong et al., 2018), SARS-CoV (Dyall et al., 2014), and SARS-CoV-2 (Jeon et al., 2020) by blocking the interaction of ACE2-Spike and Nsp14 of SARS-CoV-2 based on computational biophysics analysis (Martin and Cheng, 2020). Beck et al. used deep learning-based drug target prediction algorithm to predict that several commercially available drugs atazanavir, remdesivir, efavirenz, ritonavir, and dolutegravir potently inhibits the activity of 3CL^pro^ of SARS-CoV-2 with low Kd values (Beck et al., 2020). Gysi et al. (2020) identified 81 potential repurposing compounds against SARS-CoV-2 via graph neural network model. Zeng X. et al. (2020) used graph representation learning techniques to identify 41 potential candidates against SARS-CoV-2 infection including niclosamide, melatonin and dexamethasone.

Stebbing et al. used AI-algorithms to identify baricitinib, an approved Janus kinase (JAK)1/JAK2 inhibitor for treating rheumatoid arthritis, to be effective at inhibiting SARS-CoV-2 infection as well as reducing virus induced inflammations (Stebbing et al., 2020). Although baricitinib can cause potential adverse effect including lymphopenia, anemia and increase co-infection crisis with other pathogens (Pujari et al., 2020), its combination with remdesivir is superior to remdesivir alone in improving the clinical recovery rate of patients with COVID-19 with few serious side effects (Kalil et al., 2021). Ge Y. et al. (2021) used machine learning and statistical analysis to uncover a ploly-ADP-ribose polymerase (PARP1) inhibitor meguparib (CVL218) which reduced SARS-CoV-2 replication without toxic effects *in vitro*. In addition it also suppressed IL-6 production. Molecular docking simulation showed that meguparib can bind to the nucleoprotein of SARS-CoV-2, which might mediate the inhibition of viral replication (Ge Y. et al., 2021).

*In silico* molecular modeling suggests that several FAD-approved anticancer drugs (Capmatinib, Pemigatinib, Selpercatinib, and Tucatinib) might be able to inhibit COVID-19 by docking on M^pro^ and spike of SARS-CoV-2 (Parveen and Alnoman, 2021). Network medicine methods suggest potential drug combinations (anti-inflammatory plus anti-viral drug) for treating COVID-19, including toremifene plus emodin, mercaptopurine plus melatonin, and sirolimus plus dactinomycin (Zhou et al., 2020c). Another network medicine analysis predicted that anti-viral inhibitor toremifene plus anti-inflammatory drug melatonin have potential to treat COVID-19 (Cheng et al., 2020).

Above mentioned AI-based drugs against COVID-19 are encouraging, effective and robust experimental evaluations *in vitro* and *in vivo* will further increase the successful rate for drug discovery in preclinical and clinical trials. AI-tools also integrate pharmocogenetic and pharmocogenomic information to figure out the key genetic targets and therapies against COVID-19.

## Therapeutic Antibodies Against SARS-CoV-2

Responsible for binding the receptor and mediating cellular entry, the spike protein of SARS CoVs is the ideal target for neutralizing antibodies as therapy (Table 4). Several isolated monoclonal antibodies from SARS-CoV-2 infected patients in recovery and convalescent periods can recognize receptor-binding domain (RBD), N terminal domain (NTD) and S2 domain of spike. In one such efforts, Ju et al. (2020) isolated three potent neutralizing antibodies P2C-1F11, P2C-1A3, and P2B-2F6. The three antibodies compete with ACE2 for binding to SARS-CoV2 RBD without cross-reacting with plasma from SARS-CoV and MERS-CoV patients. Among the three antibodies, P2C-1F11 displays the most potent neutralizing activity *in vitro* and *in vivo*. It occupies a large binding surface on RBD and triggers quick and extensive shedding of spike protein from cell surface, thus neutralizes the virus (Ge J. et al., 2021). From humanized mouse model, a monoclonal antibody 47D11 was identified to bind to SARS-CoV and SARS-CoV-2 spike RBD and ectodomain with similar affinities, and the binding was not competed by ACE2 (Wang et al., 2020a). However, three reported SARS-CoV RBD-targeting mABs S230, m396, and 80R have very low cross-reactivity at different concentrations compared with SARS-CoV-2 for different antigenicity (Wrapp et al., 2020). Another reported SARS-CoV-specific antibody CR3022 derived from convalescent SARS-CoV infected patient also potently binds to RBD of SARS-CoV-2. CR3022 binds SARS-CoV-2 spike at a site that is different from the ACE2 binding site. Combination of CR3022 and other antibodies which has their epitopes in RBD, can synergistically neutralize SARS-CoV-2 (Yuan et al., 2020). Chi et al. isolated a monoclonal antibody 4A8 from convalescent patient that potently neutralizes both authentic SARS-CoV-2 and SARS-CoV-2 pseudovirus. They utilized Cryo–electron microscopy, and obtained a structure with a resolution of 3.1 angstroms for the epitope 4A8 - NTD interface (Chi et al., 2020). These results indicate that combination of RBD and NTD-targeting antibodies may be useful as therapeutic cocktails.

**TABLE 4:** Representative therapeutic antibodies strategies for CoV infections.

**Name of drugs**	**Mechanism of action**	***In vitro***	***In vivo***	**Clinical trial**	**Approved**	**References**
Meplazumab	Anti-CD147	SARS-CoV-2	SARS-CoV-2	SARS-CoV-2	N.A.	Wang et al., 2020b
P2C-1F11	Competes with ACE2 for binding to SARS-CoV2 RBD	SARS-CoV-2	SARS-CoV-2	N.A.	N.A.	Ju et al., 2020; Ge J. et al., 2021
P2C-1A3	Competes with ACE2 for binding to SARS-CoV2 RBD	SARS-CoV-2	SARS-CoV-2	N.A.	N.A.	Ju et al., 2020
P2B-2F6	Competes with ACE2 for binding to SARS-CoV2 RBD	SARS-CoV-2	SARS-CoV-2	N.A.	N.A.	Ju et al., 2020
47D11	Binds to SARS-CoV and SARS-CoV-2 spike RBD and ectodomain	SARS-CoV, SARS-CoV-2	N.A.	NCT04644120	N.A.	Wang et al., 2020a
CR3022	Binds to RBD	SARS-CoV, SARS-CoV-2	SARS-CoV-2	N.A.	N.A.	Yuan et al., 2020; Atyeo et al., 2021
4A8	Binds to NTD	SARS-CoV-2	N.A.	N.A.	N.A.	Chi et al., 2020
H4	Blocks interaction of RBD-ACE2	SARS-CoV-2	N.A.	N.A.	N.A.	Wang et al., 2020d; Wu et al., 2020c
B38	Blocks interaction of RBD-ACE2	SARS-CoV-2	N.A.	N.A.	N.A.	Wang et al., 2020d; Wu et al., 2020c
CA1	Blocks interaction of RBD-ACE2	SARS-CoV-2	SARS-CoV-2	N.A.	N.A.	Shi et al., 2020
CB6	Blocks interaction of RBD-ACE2	SARS-CoV-2	SARS-CoV-2	N.A.	N.A.	Shi et al., 2020
BD-368-2	Overlaps with the ACE2 binding site	SARS-CoV-2	SARS-CoV-2	N.A.	N.A.	Cao Y. et al., 2020
LY-CoV555	Blocks interaction of RBD-ACE2	SARS-CoV-2	SARS-CoV-2	NCT04427501	US FDA	Jones et al., 2020
REGN-COV2(REGN10933 +REGN10987)	Non-competitively bind RBD	SARS-CoV-2	SARS-CoV-2	NCT04425629 NCT04426695 NCT04452318	US FDA	Weinreich et al., 2021
JMB2002	Blocks interaction of RBD-ACE2	SARS-CoV-2	SARS-CoV-2	FDA approved for clinical trial	N/S	Gu et al., 2021

Additional potent monoclonal antibodies for specific RBD or S1 subunits were also identified *in vitro* and *in vivo* and their structures with spike or ACE2 have been deciphered (Wang et al., 2020d), these antibody includes H4, B38 (Wu et al., 2020c), CA1, CB6 (Shi et al., 2020), BD-368-2 (Cao Y. et al., 2020), and cocktail of REGN10987 and REGN10933 (Hansen et al., 2020). Still some antibodies can bind to the spike to reduce SARS-CoVs titer, but is unable to block the binding of virus and ACE2, such as n3130, n3088 (Wu et al., 2020b), and S309 from memory B cells of recovered from a patient infected with SARS-CoV, indicating multiple mechanisms of neutralizations (Pinto et al., 2020).

Clinical trials with neutralizing antibodies have been conducted extensively. In a phase II clinical trial (NCT04427501), 2,800 mg dose of SARS-CoV-2 neutralizing antibody LY-CoV555 from Lilly declined the viral load in patients (Chen et al., 2021), but LY-CoV555 in combination of remdesivir have no efficacy in hospitalized patients with COVID-19(NCT04501978) (ACTIV-3/TICO LY-CoV555 Study Group et al., 2021). A neutralizing antibody cocktail REGN-COV2 reduced viral load and enhanced immune responses (NCT04425629) (Weinreich et al., 2021). Due to the positive responses, both antibodies were authorized by US FDA for emergency use. A human anti-SARS-CoV-2 RBD antibody JMB2002, can prevent and treat SARS-CoV-2 infection in rhesus macaque by blocking the binding of ACE2 to RBD of multiple variants including the South African mutant (B.1.351), the UK mutant (B.1.1.7) and the Brazilian mutant (P.1) (Gu et al., 2021). Recently, JMB2002 was approved by the US FDA for clinical trial. To date, dozens of antibodies targeting SARS-CoV-2 spike are still undergoing clinical trials for COVID-19 treatment, and many more antibodies are in discovery research (DeFrancesco, 2020; Yang et al., 2020).

## Immuno-Regulators Against SARS-CoV-2

SARS-CoV-2 infection causes severe acute pneumonic processes with pathological damages, including inflammatory cytokine storm characterized by heightened levels of C-reactive protein, ferritin, interleukin (IL)-1, IL-6, IL-8, and TNF-α (del Valle et al., 2020; Huang et al., 2020; Moore and June, 2020; Ruan et al., 2020). Several immune interventions mitigated host organ failure in the viral pneumonia (Table 5), such as the use of corticosteroid, but there is still some debate in these uses (Russell et al., 2020; Shang et al., 2020; Zhou et al., 2020b). Supportive use of corticosteroid produced a positive effect on COVID-19 with lower mortality before the development of acute respiratory distress syndrome (ARDS) (Boglione et al., 2021). Another large-scale randomized clinical trials (NCT04381936) reported that the use of glucocorticoid dexamethasone reduced 28-day mortality of hospitalized COVID-19 patients (Recovery Collaborative Group et al., 2020). In hospitalized COVID-19 patients with symptoms of pneumonitis or hypoxia, dexamethasone or remdesivir plus baricitini have shown benefits in randomized controlled trials (Calabrese and Calabrese, 2021).

**TABLE 5:** Representative therapeutic antibodies strategies for CoV infections.

**Name of drugs**	**Mechanism of action**	***In vitro***	***In vivo***	**Clinical trial**	**Approved**	**References**
Dexamethasone	Prevents the release of substances in the body that cause inflammation	N.A.	N.A.	SARS-CoV-2	Prevents inflammation	Recovery Collaborative Group et al., 2020; Atyeo et al., 2021
Tocilizumab	Anti-IL-6	N.A.	N.A.	SARS-CoV-2	Adult patients with active rheumatoid arthritis	Stone et al., 2020
Sarilumab	Anti-IL-6R	N.A.	N.A.	SARS-CoV-2	Moderates to severe active rheumatoid arthritis	Castelnovo et al., 2021

Dampening IL6 is a potential therapeutic avenue for immuno-modulation because IL6 is elevated in patients suffering from ARDS and its level correlated positively with viral loads (del Valle et al., 2020). Tocilizumab, a monoclonal anti-IL-6 antibody, is a promising anti-inflammatory regent in the treatment of COVID-19 but the results are mixed results from clinical trials. A randomized, double-blind, placebo-controlled trial (NCT04356937) show that tocilizumab has no efficacy for improving statement of treatment in moderately hospitalized patients infected with SARS-CoV-2 (Stone et al., 2020). Tocilizumab and sarilumab, an IL-6R humanized monoclonal antibody, have been evaluated in clinical trials (NCT04330638, NCT04486521, NCT04329650) by decreasing IL6 level to decrease the risk of mortality caused by COVID-19 (Castelnovo et al., 2021).

## Discussion

In order to cope with emerging and re-emerging infectious diseases, challenges and opportunities abound for developing antiviral therapeutics. A large number of anti-viral agents are currently being explored for treating SARS-CoV-2 infection. Repurposing of existing drugs have demonstrated power by bringing several drugs to approval for treating COVID-19 patients, such as remdesivir and dexamethasone. However, these drugs still suffer from suboptimal therapeutic effect or known strong side-effect. Direct acting antivirals (DAAs) are rapidly being developed and there is great hope for producing highly potent antivirals from these efforts. Structure-based studies have significantly boosted the efficiency and precision of these developments. However, drug resistant strains are going to arise sooner or later. The recent rises of several high transmissible strains sounded alarms for currently used vaccines and neutralizing antibodies. Combinations of DAAs or neutralizing antibodies will likely be required for effective control of the viruses, much like the development of HIV cocktail therapies. On the other hand, development of broad-spectrum antiviral drugs not only fight against COVID-19 but also provide arsenals for protection from future viral outbreaks. The wealth of knowledge that has been and continues to be gleaned on the interaction between viruses and hosts will guide and prod the development of host-targeting antivirals, which have the potential advantages of broad-spectrum therapeutic effect and insensitivity to viral evasion. Antiviral drug development in general will benefit from the heightened level of social awareness of the COVID-19 epidemic and hopefully provide a safeguard against future viral epidemics for the humankind.

## Author Contributions

MM and XT wrote the manuscript. Both authors contributed to the article and approved the submitted version.

## Conflict of Interest

The authors declare that the research was conducted in the absence of any commercial or financial relationships that could be construed as a potential conflict of interest.
